# Parent- and teacher-reported long-term effects of parent training on child conduct problems in families with child protection and other support services: a randomized controlled trial

**DOI:** 10.1186/s13034-021-00358-6

**Published:** 2021-02-11

**Authors:** Piia Karjalainen, Päivi Santalahti, Eeva T. Aronen, Olli Kiviruusu

**Affiliations:** 1 Department of Public Health Solutions, Mental Health Unit, Finnish institute for health and welfare, P.O. Box 30, 00271 Helsinki, Finland; 2 Department of Child Psychiatry, Sörnäinen Child Psychiatry Outpatient Clinic, Helsinki University Hospital, P.O. Box 605, 00029 HUS Helsinki, Finland; 3 Department of Child Psychiatry, University of Turku, P.O. Box 52, 20521 Turku, Finland; 4 Department of Child Psychiatry, Children’s Hospital, Helsinki University Hospital and University of Helsinki, PL 347, 00029 HUS Helsinki, Finland; 5 Laboratory of Developmental Psychopathology, Child Psychiatry, Helsinki Pediatric Research Center, University of Helsinki and Helsinki University Hospital, Tukholmankatu 8 C 613, 00290 Helsinki, Finland

**Keywords:** Parenting program, Child behavior problem, Child protection services, Evidence-based program, Long-term effectiveness, Incredible years

## Abstract

**Background:**

This randomized controlled trial (RCT) evaluated the long-term effectiveness of the Incredible Years^®^ (IY) Parenting Program in modifying children’s externalizing problems among families in Child Protection Services (CPS) and using other special support services. We also examined whether parent-reported effects of the IY^®^ generalize to the daycare/school setting as reported by teachers.

**Methods:**

Participants in the study were 3–7-year-old children with behavioural problems (*N* = 102 at baseline, *N* = 89 at one-year follow-up). Participants were randomized to intervention (*N* = 50) and control groups (*N* = 52) after the baseline assessment. The intervention group received 19-week IY^®^ Parenting Program. The effectiveness of the intervention was analyzed using linear mixed model.

**Results:**

Our previously reported pre-post intervention effects on CBCL (Child Behavior Checklist) and ECBI (Eyberg Child Behavior Inventory) were not sustained to the one-year follow-up. Child conduct problems decreased from baseline to follow-up in both intervention and control groups. The positive changes were not observed at daycare/school from baseline to post-intervention or to the one-year follow-up, and there were no significant differences in changes between the groups.

**Conclusions:**

Evidence-based parenting program IY^®^ seems to be an effective intervention for child conduct problems in the short term in families in the CPS context, but sustaining the positive effects and generalizing them to the daycare/school context are challenging.

*Trial registration*: The trial is registered in the ClinicalTrials.gov registry (NCT03239990), Registered August 4th, 2017; https://clinicaltrials.gov/ct2/results?cond=&term=NCT03239990&cntry=&state=&city=&dist=

## Introduction

Conduct disorder and conduct problems are prevalent among children with Child Protection Services (CPS) contact [1]. Epidemiological studies have shown that the world-wide prevalence of conduct disorders is 5.7% in the general population [2], and almost four times higher among children with CPS contact [1]. However, children within these services often do not receive adequate psychiatric help [3–5] tailored to meet their complex and specific needs [6]. According to Vinnerljung et al. [7], children in CPS are five to eight times more likely to have been hospitalized for serious psychiatric disorders in their teens and four to six times more likely in young adulthood than their peers. For this reason, there is an alarming need for interventions to reduce children’s conduct problems in families with CPS contact. However, there is surprisingly little research investigating effective means to help these families.

Children with early externalizing problems have a higher risk of developing later problem behaviours such as substance abuse and criminal and violent behaviours. Externalizing problems also predict mental disorders, poor health, academic underachievement, unemployment, family problems and increased mortality in adulthood [8–12]. Children with conduct problems exhibit often multiple risk factors in their lives such as child maltreatment, harsh parenting, lack of parental involvement and sensitivity, disrupted families and low family income [13]. Behavioural disorders cause high costs to society in service use and benefits, physical and mental hospitalization, special education and criminal justice [14–16]. The families of these children are quite often clients of CPS and other social services. Parenting practices have been found to be suboptimal in families with CPS contact and may increase child problem behaviour [17]. Parenting practices may mediate the transmission of risk behaviours across generations [18]. It is important to examine whether parent management interventions for treatment of children’s problem behaviours work among families in the most vulnerable situations.

Systematic reviews and meta-analyses have shown that parent training programs, including Incredible Years^®^ (IY), are one of the most effective methods to reduce children’s conduct problems [19, 20]. However, less is known about how effective they are in special groups, such as among families with CPS contact, or how the positive intervention effect is sustained over time. Some reviews have reported the long-term effects from pre-intervention to follow-up [17, 21–24], showing effect stability, but there is considerable heterogeneity within the results. The long-term RCT studies of IY^®^ (six months to two years) showed, in eight out of ten studies, positive effects for child externalizing behaviours in the intervention group compared with controls [25–32]. The positive effects were also seen 5.6–10.5 years after the intervention [33]. However, no studies have evaluated the long-term effects of parent training programs in the actual CPS context, although Hurlburt et al. [34] noted that parents with a history of maltreatment in the IY^®^ group, compared with parents in the control group, reported more positive changes in child behaviour at the one-year follow-up.

Up to 83% of children who experience clinical levels of conduct problems at home also experience them in daycare or school settings [35]. These children are likely to be not only more aggressive but also have impaired social skills such as lack of prosocial and positive communication skills required to be able to interact in a group [36, 37]. They also have self-regulation problems and non-compliance [38], which lead to problems interacting with peers and teachers [39–41]. Furthermore, academic difficulties, such as lower reading and math skills during childhood, and general impaired cognitive functioning are common among children in families with CPS contact [42–45], with a higher risk of school drop-out [46], grade retention and suspension [47]. Poor academic performance further increases the risks for later antisocial behaviours, poor health and incarceration [48].

In parent training literature, there have been relatively few studies examining how the effects of parenting programs are shown in daycare or school environment. In the few existing studies, findings have been contradictory. Some studies have found improvements in child behaviour in daycare/school through parent training [49, 50], while others have not found the generalization effect [51–54]. None of the earlier studies conducted within the CPS context have examined how parent training affects children’s behaviour in other environments such as daycare and school.

Although numerous RCTs have reported short-term results and some also long-term effectiveness of parenting programs in reducing child externalizing symptoms, Barlow and Coren [17] suggest in their review of Campbell Reviews that further research is needed to assess the effectiveness of parent training programs for specific subgroups of parents in addition to their long-term effectiveness. The lack of quality research on child externalizing symptoms in specific subgroups, such as children involved in CPS, was also found in our systematic literature review [55]. Since parents and children involved in CPS exhibit more challenges and risk for child conduct disorders than the populations usually examined, it is important to know whether intervention effects of parenting programs are maintained at follow-up.

To address these gaps in knowledge, we have conducted an RCT on the effects of the IY^®^ Parenting Program intervention among clients in CPS and other social support services. Since we have already reported the results from pre- to post-intervention based on parental reports [56], the aim of the present study was to examine within the same RCT condition 1) whether the effects of the IY^®^ Parenting Program on child externalizing behaviour observed from pre- to post-intervention are sustained at the one-year follow-up as reported by parents, and 2) whether the effects of the IY^®^ on child externalizing behaviours observed by parents at home generalize to daycare/school settings as reported by teachers.

## Materials and methods

### Study design and procedure

This is a Randomized Controlled Trial (RCT) on the effectiveness of the IY^®^ Parenting Program conducted in families receiving child protection and other family support services. The study protocol has been described in detail in Karjalainen et al. [56]. The study ran from 2015 to 2017 with a one-year follow-up in 2018, and was carried out in seven municipalities, mainly in the southern part of Finland, with originally 102 children (aged 3–7 years) with behaviour problems and their parents (*N* = 122) attending the study. Participants were assigned randomly to IY^®^ (*N* = 50) and control group (*N* = 52). Intervention groups began in fall 2016 or winter 2017. There were three measurement points: at baseline (pre-intervention; done before random allocation of participants), three months after the intervention (post-intervention) and at 12 months after the intervention (one-year follow-up). In addition to parental reports, data were also collected from daycare/school teachers at these same measurement points.

This study comprises parent-reported follow-up data from pre- and post-intervention to the one-year follow-up and teacher-reported data from pre- to post-intervention, from post-intervention to the one-year follow-up and from pre-intervention to the one-year follow-up. The present study focuses on the primary outcomes of the RCT, i.e. measures of child behavior problems.

### Ethics approval

The study was approved by the Intermunicipal Hospital District of Helsinki-Uusimaa Ethics Committee in February 2016, and the trial is registered in the ClinicalTrials.gov registry (NCT03239990).

### Participants

The participants were 3–7-year-old children with behavioural problems and their parents. Families were currently clients of CPS or clients of social services indicated to need support in parenting.

In Finland, CPS consists of preventive CPS (e.g. strengthened in-home family help, family counselling, parenting groups), non-institutional care at home and institutional care (e.g. emergency placement of the child or child living in foster home or children's home). This study contained only families who received preventive CPS and non-institutional care.

**Fig. 1:**
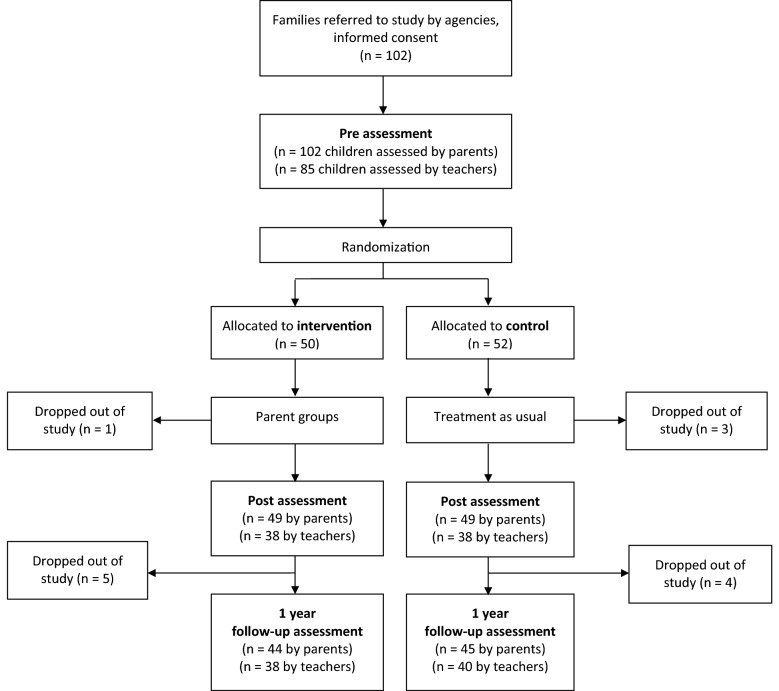
Participant flowchart

Parents whose children were living at home, who were motivated, who were able to participate and who were assumed to benefit from the program after being assessed by social and family workers were taken into the study. Parents’ participation was declined if an acute child protection issue was unresolved (child’s basic needs and safety not met) or if the parents had a mental health or substance abuse problem that prevented them from attending the intervention. Parents in the control group were entitled to use all other services except the group-based IY^®^ Parenting Program, which they were able to access after the study. For participation in the study, the participants received a gift certificate to a swimming pool, cinema or activity park after each assessment. Of the original 102 children, parent-reported data were available for 98 and 89 children at post-intervention and the one-year follow-up, respectively. Teacher-reported data were available for 85 children at pre-intervention, 76 children at post-intervention and 78 children at the one-year follow-up. Figure 1 presents the participant flowchart.

### Intervention

Participants attended IY^®^ Preschool BASIC Parenting Program [57], with 19–20 group meetings supported by four additional, structured and individualized 1- to 1.5-h sessions of IY^®^ Home Coaching to enhance learning, not usually included in the program.

The goal of the intervention was to improve child behaviour by improving parent–child interaction, enhancing positive parenting skills, decreasing harsh and abusive parenting, increasing parental involvement and sensitivity and improving knowledge of child development. Parents are taught to use more positive, consistent strategies for reducing child misbehaviour by watching DVDs, rehearsing and having group discussions. The way of working with parents is highly collaborative and interactive and takes different learning styles into account. The group leaders do not act from an expert role but are part of an active, self-reflective, reciprocal process that utilizes the knowledge, strengths and views of both the group members and group leaders equally. Furthermore, parents set their individual goals to work towards, and their successes in reaching these goals are celebrated.

The groups consisted of 10–12 parents who met weekly for about two hours at a time. Each group had three trained group leaders, two of whom were from family counselling services and one from CPS, who also conducted the family visits. All had been trained as IY^®^ BASIC Group Leaders and IY^®^ Home Coaches. The group leaders followed the structured manual and filled out self-evaluations and checklists after each group meeting to ensure program fidelity. They also received supervision and attended one full-day coaching session run by a certified IY^®^ trainer. Evaluations from the parents after each group meeting and a final evaluation after completion of the program were also gathered.

### Measures

The *Family Basic Demographic questions* included background information on the children, the mothers and fathers (age, marital status, education) and their family circumstances, i.e. unemployment, financial worries and major incidents affecting the family.

#### Parent-reported child behaviour measures

*Eyberg Child Behavior Inventory* (ECBI) is a validated, reliable and widely used measure to assess child problem behaviour reported by parents [58]. It consists of two scales, the *Intensity Scale* and the *Problem Scale*, which elicit parents’ perceptions of 36 problem behaviours. The *Intensity Scale* consists of a seven-point Likert-type scale (never to always) that measures the frequency of the problem behaviours, while the *Problem scale* measures whether or not the parent sees the particular behaviour as a problem by eliciting a yes–no answer. Internal consistency (alpha) of the 36 items at baseline was 0.91 and 0.87 for the *Intensity Scale* and the *Problem Scale*, respectively.

*Child Behavior Checklist (CBCL)—Parent Report Form* is another widely used measure with good reliability and validity [59]. It consists of 99 emotional and behavioural statements rated by parents. In this study, we used the CBCL’s 24-item *Externalizing Scale* and its subscales of *Attention Problems* (five items) and *Aggressive Behaviour* (19 items). The items are rated on a three-point Likert-type scale (not true to very true/often true). Internal consistency for the 24-item *Externalizing Scale* was 0.89 at baseline.

#### Teacher-reported child behaviour measures

Teacher-reported SESBI-R, equivalent to parent-reported ECBI, consists of 38 items that measure the frequency (*Intensity Scale*) and severity (*Problem Scale*) of behaviour problems [58]. The behaviours are rated on a seven-point Likert-type scale ranging from never to always (*Intensity Scale*) and a dichotomous yes/no format (*Problem Scale*). Internal consistency (alpha) of the 38 items was 0.97 for the *Intensity Scale* and 0.96 for the *Problem Scale* at baseline.

Caregiver-Teacher Report Form (C-TRF) and Teacher Report Form (TRF) evaluate emotional and behavioural problems that a child may display in kindergarten/daycare or at school [59]. The items are rated on a three-point Likert-type scale (not true to very true/often true). C-TRF is used by teachers of 1½–5-year-old children and TRF for 6–18-year-old children. In this study, we used the *Externalizing Scale* and its subscales. When calculating the scores, we used only those items that were equivalent between C-TRF and TRF, seven items in *Attention Problems Scale* and 17 items in *Aggressive Behaviour Scale.* Internal consistency for the *Attention Problems Scale* was 0.91 and for the *Aggressive Behaviour Scale* 0.95*.*

### Statistical methods

The unit of analysis was a child (or a reporting parent). If two parents participated and reported data, we used the answers of the mother.

To analyse intervention effectiveness, we used linear mixed models to take into account clustering of observations due to intervention group/municipality and due to repeated measurements of the same subjects. In the analyses, it turned out, however, that the intervention group/municipality level did not produce any significant variance in the outcomes, and thus, this level was omitted from the final analyses. From the mixed models, estimated marginal means and their standard errors were obtained for different time points and study groups (intervention vs. control). T-tests were used to analyse within-group changes in the measures of child problem behaviour. From these same mixed models, also the following contrasts were tested to answer the study questions: difference in outcome changes between the study groups (1) from pre- to post-intervention, (2) from post-intervention to follow-up and (3) from pre-intervention to follow-up. In addition to the intent-to-treat analyses, per protocol analyses were carried out. In these, only those children were included in the intervention group, whose parents had attended at least nine (out of 19) intervention group sessions. Effect sizes were presented using Cohen’s d, which were calculated from difference scores (pre-post, post-follow-up and pre-follow-up) in order to account for possible baseline differences between the study groups. Alpha level of 0.05 for p values was used to indicate statistical significance. All analyses were conducted using SPSS software version 26.

## Results

The participating children were mainly boys (N = 65/102). The mean age of children was 5.3 years, and they were almost all Finnish-speaking (97.1%). Almost half of the parents (46.1%) were single. Most of the parents (83.3%) had difficulties in covering expenses with their current income. In one-third of the families (31%), there had been three or more stressful events during the last 12 months, e.g. divorce, unemployment, serious illness or death of a family member. Almost one-fifth of participating mothers (17.3%) had no vocational education, and almost half (41.8%) were not employed at baseline. Most of the fathers (70.8%) had intermediate education, and most (79.2%) were employed. Both mothers and fathers (49%) were mostly between 30 and 39 years of age. The socio-demographic characteristics of the intervention and control groups did not differ significantly at baseline. The socio-demographic characteristics of the families are presented in more detail in Karjalainen et al. [56].

### Changes in outcomes within groups

Both groups showed significant improvements in all domains of ECBI and CBCL from pre- to post-assessment, except in CBCL aggressive behaviour in the control group (Table 1). No significant changes occurred between post-assessment and the one-year follow-up in either group in any domain, other than an increase in ECBI Problem scores in the intervention group. Both groups showed significant improvements in all domains of ECBI and CBCL from pre-intervention to the one-year follow-up. No significant improvements occurred between time points in any of the domains of SESBI-R or TRF in either the intervention or control group.

**Table 1:** Mean (SE) changes in measures of child problem behavior within groups between different time points

Measure	Mean (SE) change^a^								
	Pre-post			Post-follow-up			Pre-follow-up		
	Mean (SE)	t Test	*p*	Mean (SE)	t Test	*p*	Mean (SE)	t Test	*p*
*Parent report*									
ECBI									
Intensity									
IY	18.8 (3.3)	5.7	< 0.001	− 5.8 (3.7)	− 1.6	0.120	12.9 (3.7)	3.5	0.001
Control	12.8 (3.5)	3.6	0.001	4.5 (5.0)	0.9	0.374	17.3 (4.3)	4.1	< 0.001
Problem									
IY	8.6 (1.0)	8.7	< 0.001	− 2.3 (1.1)	− 2.2	0.038	6.3 (1.2)	5.1	< 0.001
Control	2.8 (1.2)	2.3	0.024	2.8 (1.5)	1.9	0.060	5.7 (1.3)	4.4	< 0.001
CBCL									
Attention problems									
IY	0.9 (0.2)	3.5	0.001	− 0.2 (0.2)	− 1.1	0.265	0.6 (2.7)	2.4	0.020
Control	0.7 (0.2)	2.6	0.011	0.1 (0.2)	0.2	0.832	0.7 (0.2)	3.1	0.003
Aggressive behaviour									
IY	4.4 (0.9)	4.7	< 0.001	− 0.9 (0.7)	− 1.2	0.222	3.5 (1.0)	3.5	0.001
Control	2.0 (1.0)	2.0	0.054	2.1 (1.2)	1.7	0.091	4.1 (1.1)	3.9	< 0.001
*Teacher report*									
SESBI-R									
Intensity									
IY	3.8 (5.2)	0.7	0.474	1.6 (6.4)	0.2	0.804	5.4 (6.3)	0.8	0.401
Control	10.2 (5.2)	2.0	0.054	− 9.6 (6.6)	− 1.5	0.155	0.6 (6.5)	0.1	0.927
Problem									
IY	1.6 (1.4)	1.2	0.258	− 1.5 (1.7)	− 0.9	0.396	0.1 (1.6)	0.7	0.943
Control	− 0.5 (1.2)	− 0.4	0.677	− 1.9 (1.6)	− 1.2	0.257	− 2.4 (1.7)	− 1.4	0.173
TRF									
Attention problems									
IY	0,3 (0.4)	0.7	0.479	0.7 (0.5)	1.3	0.197	1.0 (0.5)	1.9	0.067
Control	0.5 (0.5)	1.0	0.308	− 0.0 (0.5)	0.06	0.955	0.5 (0.5)	1.1	0.287
Aggressive behaviour									
IY	− 0.1 (0.9)	− 0.1	0.920	0.7 (1.2)	0.6	0.554	0.6 (1.3)	0.5	0.622
Control	1.3 (0.8)	1.7	0.105	− 0.7 (0.8)	− 0.9	0.401	0.7 (1.1)	0.6	0.523

*ECBI* Eyberg Child Behavior Inventory, *CBCL* Child Behavior Checklist, *SESBI-R* Sutter-Eyberg Student Behavior-Inventory Revised, *TRF* Teacher Report Form

^a^Estimated marginal means

Baseline differences between the groups in child behavior problems were also tested: there were no statistically significant differences in any of the studied domains of child problem behavior (ECBI, CBCL, SESBI-R and TRF) at baseline (all p > 0.15).

### Intervention effectiveness—parent-reported outcomes

**Fig. 2:**
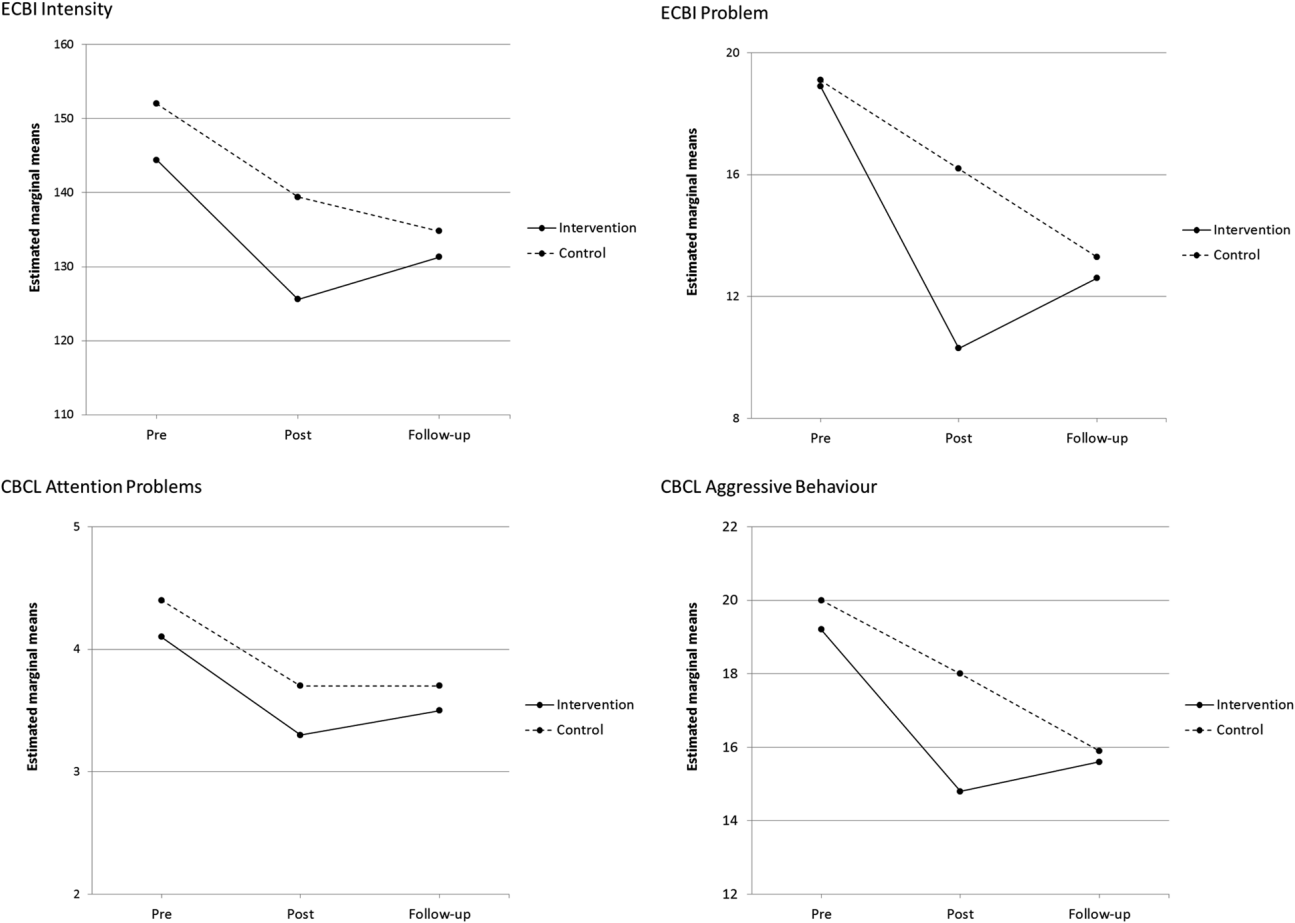
Parent-reported results

Results for intervention effectiveness regarding parent-reported outcomes from pre- to post-intervention have been reported earlier in Karjalainen et al. [56] and are presented here only for the sake of clarity. From pre- to post-intervention, parent-reported results showed that the intervention group had a larger reduction in *ECBI* scores over time than the control group, but the reduction was statistically significant only on the *ECBI Problem Scale.* No statistically significant differences emerged in changes on the *CBCL* scales from pre- to post-intervention between the groups (Table 2 and Fig. 2).

**Table 2:** Estimated marginal means (and standard errors) of child outcomes pre-intervention, post-intervention and follow-up by study group and estimated intervention effects between different time points from linear mixed models

Measure				Difference in change between intervention and control groups								
	Pre	Post	Follow-up	Pre-post			Post-follow-up			Pre-follow-up		
	M (SE)	M (SE)	M (SE)	Estimate (95% CI)	*p*	d^a^	Estimate (95% CI)	*p*	d^a^	Estimate (95% CI)	*p*	d^a^
*Parent report*												
ECBI												
Intensity												
IY (*N* = 44–49)	144.4 (3.8)	125.6 (4.2)	131.3 (4.7)	6.1 (− 3.4 to 15.6)	0.204	0.26	− 10.2 (− 22.5 to 2.0)	0.101	− 0.51	− 4.1 (− 15.2 to 7.0)	0.466	− 0.16
Control (*N* = 45–48)	152.0 (3.8)	139.4 (4.2)	134.8 (4.7)									
Problem												
IY (*N* = 42–45)	18.9 (1.2)	10.3 (1.3)	12.6 (1.4)	5.8 (2.7 to 8.9)	< 0.001	0.82	− 5.3 (− 8.9 to − 1.6)	0.005	− 0.90	0.6 (− 3.0 to 4.2)	0.755	0.11
Control (*N* = 38–40)	19.1 (1.2)	16.2 (1.3)	13.3 (1.5)									
CBCL												
Attention problems												
IY (*N* = 44–49)	4.1 (0.3)	3.3 (0.3)	3.5 (0.3)	0.2 (− 0.5 to 0.9)	0.557	0.11	− 0.3 (− 0.9 to 0.4)	0.394	− 0.24	− 0.1 (− 0.8 to 0.6)	0.838	− 0.05
Control (*N* = 45–49)	4.4 (0.3)	3.7 (0.3)	3.7 (0.3)									
Aggressive behavior												
IY (*N* = 44–49)	19.2 (1.1)	14.8 (1.0)	15.6 (1.1)	2.4 (− 0.4 to 5.1)	0.089	0.34	− 2.8 (− 5.6 to 0.0)	0.052	− 0.50	− 0.4 (− 3.3 to 2.5)	0.777	− 0.08
Control (*N* = 45–49)	20.0 (1.1)	18.0 (1.1)	15.9 (1.1)									
*Teacher report*												
SESBI-R												
Intensity												
IY (*N* = 37–40)	124.2 (7.5)	120.4 (7.7)	118.8 (8.6)	− 6.5 (− 21.1 to 8.1)	0.380	− 0.25	11.3 (− 7.2 to 29.7)	0.227	0.34	4.8 (− 13.3 to 22.8)	0.600	0.08
Control (*N* = 37–45)	131.4 (7.3)	121.1 (7.6)	130.8 (8.5)									
Problem												
IY (*N* = 32–39)	9.7 (1.7)	8.2 (1.7)	9.6 (2.0)	2.1 (− 1.6 to 5.9)	0.252	0.20	0.5 (− 4.3 to 5.2)	0.839	0.09	2.6 (− 2.1 to 7.4)	0.271	0.23
Control (*N* = 34–43)	11.6 (1.6)	12.2 (1.7)	14.2 (2.0)									
TRF												
Attention problems												
IY (*N* = 38–40)	6.4 (0.6)	6.1 (0.6)	5.4 (0.7)	− 0.3 (− 1.6 to 0.9)	0.605	− 0.11	0.7 (− 0.7 to 2.1)	0.319	0.23	0.4 (− 1.0 to 1.8)	0.585	0.09
Control (*N* = 38–45)	7.0 (0.6)	6.5 (0.6)	6.5 (0.7)									
Aggressive behavior												
IY (*N* = 38–40)	10.3 (1.4)	10.5 (1.3)	9.7 (1.6)	− 1.3 (− 3.8 to 1.1)	0.283	− 0.22	1.3 (− 1.7 to 4.3)	0.400	0.31	− 0.5 (− 3.3 to 3.2)	0.970	− 0.02
Control (*N* = 38–45)	12.8 (1.4)	11.6 (1.3)	12.1 (1.5)									

*ECBI* Eyberg Child Behavior Inventory, *CBCL* Child Behavior Checklist, *SESBI-R* = Sutter-Eyberg Student Behavior Inventory–Revised, *TRF* Teacher Report Form

^a^Effects sizes: Cohen’s d calculated using difference scores between time points; 0.2 = small, 0.5 = medium, 0.8 = large effect.

From post-intervention to the one-year follow-up, there was a decrease in the control group and an increase in the intervention group in how problematic parents perceived their child’s behaviour to be, as measured by the *ECBI Problem Scale*, and this difference between groups was statistically significant (*p* = 0.005, d = − 0.90). There was a similar pattern of changes between the groups on the *CBCL Aggressive Behaviour Scale* and on the *ECBI Intensity Scale,* albeit not statistically significant. Only minor changes occurred on the *CBCL Attention Problems Scale* from post-intervention to follow-up (Table 2 and Fig. 2).

Children’s problematic behaviour on all domains in both the intervention and the control group showed improvement from pre to follow-up measurement. However, the changes in scores between groups did not differ statistically significantly (Table 2 and Fig. 2).

### Intervention effects—teacher-reported outcomes

**Fig. 3:**
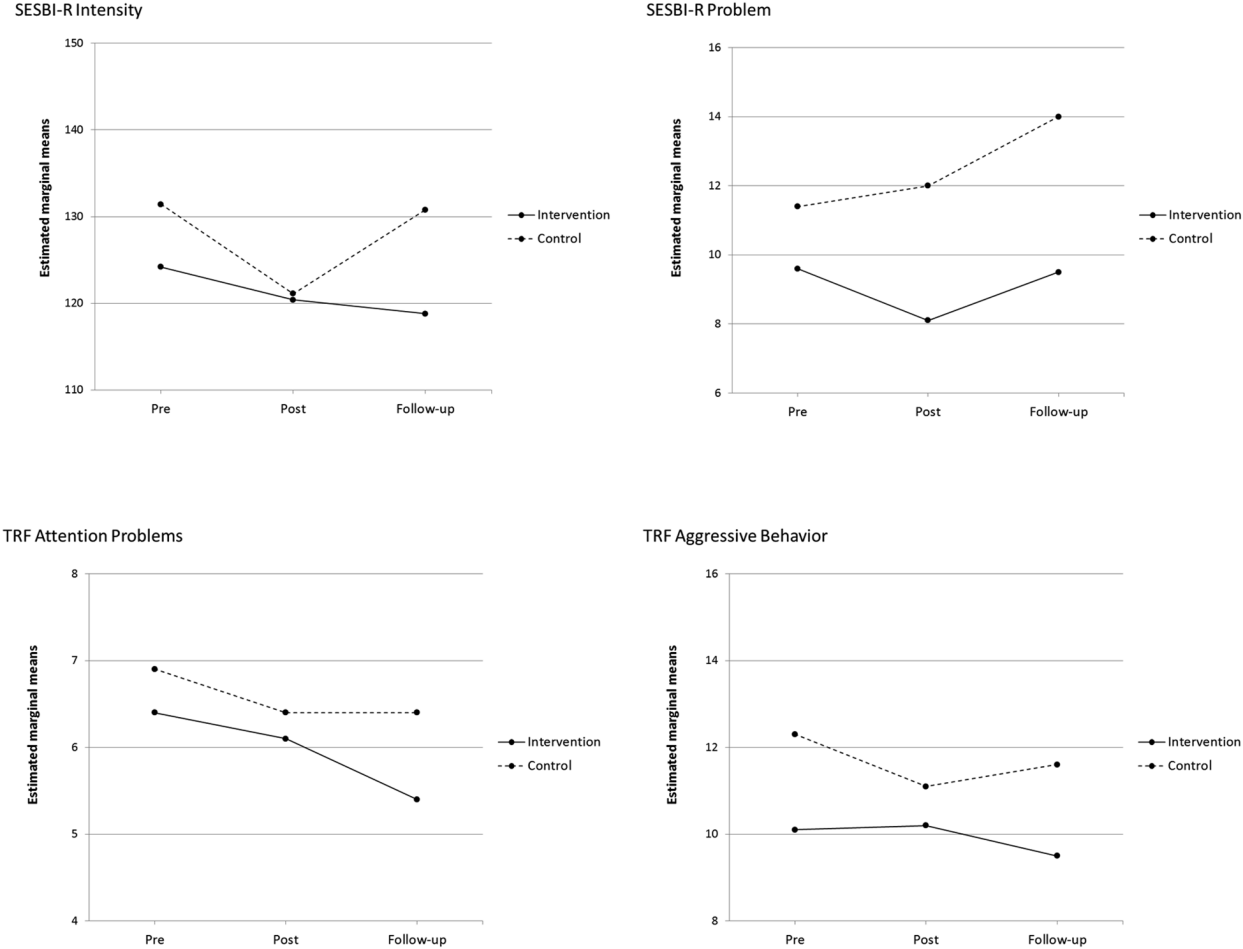
Teacher-reported results

When comparing the teacher-reported assessments, pre- and post-intervention changes in scores were in favour of the control group in all but one domain (*SESBI-R Problem Scale*), but none of the differences in changes between the groups were statistically significant (Table 2 and Fig. 3).

From post-intervention to follow-up, all changes in scores were in favour of the intervention group, but the differences between groups were not statistically significant and the effects were small, the strongest (d = 0.34) being on the *SESBI-R Intensity Scale* (Table 2 and Fig. 3).

From pre-intervention to the one-year follow-up measurement, there were small reductions on all scales, except the *SESBI-R Problem Scale,* in both groups. Changes were mostly in favour of the intervention group, but statistically non-significant and the effects were small (d < 0.25) (Table 2 and Fig. 3).

### Intervention effects—per protocol analyses

Intervention effects were also analyzed with only those children of the intervention group included whose parents had attended nine or more group sessions. In these per protocol analyses (results not shown) the results remained essentially the same as in the intent-to-treat analyses presented in Table 2.

## Discussion

The two aims of the study were to assess the long-term effects of the IY^®^ Parenting Program on child externalizing symptoms in the CPS context, and to evaluate whether parent-reported positive changes in child problem behaviours can be seen in daycare/school settings immediately after the parenting intervention and after one year. Although parents continued to report reduced levels of child behaviour problems in the intervention group, the reduced levels reported immediately after the intervention were not sustained at one year, but were still significantly lower than at baseline. The child problem behaviour levels in the control group diminished steadily from baseline to follow-up, being close to the levels reported by parents in the intervention group. The positive changes in the levels of child behaviour problems observed at home and reported by parents were not observed at daycare/school from baseline to post-intervention or to the one-year follow-up, and there were no significant differences in changes between the groups. The study suggests that evidence-based parenting program IY^®^ can reduce child behaviour problems in the CPS context. However, the positive effects seem to be more evident immediately after the intervention, and sustaining them over time or generalizing them to the daycare/school context is challenging.

As was reported in our earlier paper [56], children in the intervention group demonstrated significant positive changes on the *ECBI Problem Scale* from pre- to post-intervention compared with children in the control group. In the present study, looking at the change from post-intervention to follow-up, the behaviour of control children continued to improve, whereas the behaviour of children in the intervention group regressed slightly. These differences in changes between groups were statistically significant. Thus, looking at the changes from baseline to the one-year follow-up, the intervention effects on the *ECBI Problem Scale* over the short term were not sustained. The same pattern of changes was noted on the *CBCL Aggressive Behaviour* and also on the *ECBI Intensity* and *CBCL Attention Problems*, although the changes in the latter two groups were not statistically significant. Furthermore, the difference in scores between the intervention and control groups at follow-up seemed negligible. Importantly, however, the child problem behaviour decreased in both groups from pre-intervention to follow-up.

Our results are in line with another RCT study conducted among families reporting a history of child maltreatment [34], in which child behaviour problems first decreased (pre-post) and then increased again slightly in both conditions. However, our results contradict the findings from other previous IY^®^ studies, in which the majority showed sustained intervention effects at 6–12 months (e.g. [29–31]). Only two studies did not find a sustained effect: one with improvement at 12-month follow-up for all intervention groups (IY^®^ and minimal book intervention), but no overall treatment group effects [28], and the other among a high-risk, ethnically diverse population 60]. However, the results from these studies are not fully comparable to ours since none of them were conducted in the CPS context.

There might be a few explanations for why the intervention effects did not remain. One reason might be that since the target group probably has multiple challenges in everyday life, the intervention group might have served as a strong, positive peer support network giving its members the strength to face everyday challenges and problems with the child. Added to this, the group leaders celebrated successes and used a strengths-based approach in their work with parents. Moreover, parents were helped to learn new skills, and they were supported in trying them out. After the intervention, it is likely that most of the other challenges in their lives remained, and without support from the group and the group leader, parents started to go back to their negative ways and thinking. This might have had an impact on how parents viewed and rated their child’s behaviour. Even though child behaviour might have been better at the one-year follow-up, parents might have experienced the behaviour more negatively than it actually was, perhaps because of other ongoing stressors in their lives. This speculation is supported by teacher reports from daycare and school suggesting that the children’s behaviour continued to improve (albeit non-significantly), especially in the intervention group.

Furlong and colleagues [61] conducted a qualitative study on long-term experiences of parent training. They found out links between relapse in child behavior and abandoning learned skills in stressful times, unsupportive environment and the perceived ineffectiveness of parenting skills. Furthermore, maintaining positive results were also associated with flexibility in the implementation of skills despite difficulties and the use of available social supports. It seems that in order to make the intervention effects last in the intervention group, parents might have benefitted from longer term support to remind them of the effective positive parenting practices and help them to maintain a positive attitude. This group of families faces many stressors that limit their possibilities to integrate and generalize their newly acquired skills in daily life and it is easy to fall back on old habits. In order to make the long-term effect more sustainable, continuing contact with support groups, adding group booster meetings over a longer period of time, or delivering a shorter home coaching program after the end of the intervention might help to facilitate continued parental sense of gains (see e.g. Stewart-Brown et al. [32]. It would be important to consider implementing these modalities of continuing support in parenting interventions delivered in CPS context.

Another reason for the non-sustained positive effects might be that in Finland adult mental health and family support is considered to be of high quality and reasonably accessible, and CPS places a strong emphasis on family support and preventive work. In Finland, CPS provides strengthened in-home family help, family counselling and parenting groups for parents as part of preventive services and also part of actual CPS services. When at the post-treatment measurement parents were asked about their use of services, parents in the control group had 70 mentions of use of the different forms of support for parents (meetings with CPS workers, CPS home support service, family counselling clinic, maternity clinic, mental health clinic, adult psychiatry, psychologist meetings, therapy, couples therapy, support families, professional rehabilitation) and 17 mentions of support given directly to the child (child psychiatry, child neurologist, child psychologist, speech therapy, occupational therapy) (results not shown). Furthermore, since some of the social workers and family counselling workers in the research cities had undergone IY^®^ training, they most likely also used the same methods and principles when working with parents in the control group, who were their clients. For this reason, the control group might have been somewhat contaminated, no longer serving as a true control.

In our study, we found no signs that the few positive findings reported by parents had generalized to the school context. Our findings are in line with some other parent intervention studies on IY^®^ [52, 53, 62], on PCIT [51], on Triple-P [54] and on Rational Positive Parenting Program [63], where no significant differences (and thus, no generalization effects) were found between the groups from pre- to post-assessment or follow-up. These studies used multiple informants, i.e. parents and teachers. However, none of these studies were conducted in the CPS context. The findings of these studies contradict the findings from some other studies, e.g. another RCT study of IY^®^ conducted in the normative population [26], in which high-risk classroom behaviour problems relative to controls improved significantly from pre- to post-assessment. However, at the one-year follow-up of that study, most children in the high-risk classroom behaviour problem groups had improved regardless of the intervention condition. Similar pre-post effects were seen in a PCIT study by Funderburk [64], in a PMT study by Braet et al. [65], in a PMTO study by Kjøbli et al. [49] and in a PCIT study by McNeil et al. [50], in which children with severe conduct problems displayed significantly greater improvements than controls. However, these studies showed heterogenic results at the one-year or 18-month follow-ups regarding sustained effects of positive behaviour change. Nevertheless, our results from CPS context and with no generalization effects to daycare or school suggest that it would be important to support children also in their other growth environments, and thus provide e.g. teachers in daycares/schools, tools to facilitate child behavior change.

Our study, being one of the rare RCT studies on parenting programs conducted in a real-life setting in CPS, raises a number of suggestions for future research. First of all, it is essential to measure use of other services obtained by treatment and control groups during the CPS involvement in order to control for their effects in the analyses. When doing longitudinal research in daycares/schools, it would be necessary to minimize changes in informants (teachers) to reliably detect changes in child’s behavior. Also, including observational data on child behavior would help to better understand the differences between parent and teacher ratings. To facilitate generalization effects to school context, adding a training component for teachers in managing child misbehavior to intervention (e.g. IY Teacher Management Training) would be worth examining. Furthermore, since behavior problems are likely to affect school achievement, measures of child’s academic performance (e.g. math and writing/reading skills) should be included as one of the main outcomes. In future studies, examining the effect of parent training also on internalizing symptoms would be interesting, especially among young children, whose behavioral profiles are not fixed and both externalizing and internalizing problems may be present [66].

## Strengths and limitations

Our study has several strengths as well as some limitations that should be noted. The main strength of the study is its strong RCT design and the fact that this is one of the first RCTs conducted among families involved with CPS that focused on children living at home. Furthermore, due to researcher’s persistent efforts to contact the participants of the study, we were able to reach almost all of the participants at post-measurement and at follow-up. To examine sustained effects, we also included a control group in the one-year follow-up measurements. We used reliable, validated and internationally widely used measures to evaluate child problem behaviours. The use of multiple informants increases the reliability of the results. Since this research was done in a multicentre, non-clinical environment, it is more reflective of real-world clinical practice.

The smaller-than-calculated sample size is the biggest limitation of the study. The loss of power affected our ability to find significant differences. Even though we used multiple informants, we did not use observational evaluation, which would have further increased the reliability of our results. Since some children moved on to other grades or daycare centres or from daycare to school during the study period, the same teachers did not necessarily answer the questionnaires at different time points. Furthermore, our study sample was heterogeneous, including also non-CPS clients. This should be taken into consideration when comparing our results to studies with exclusively CPS clients. Quality and accessibility of support services in Finland are good. For this reason, parents in the control condition received good care and services from health and social services and from their child-care facilities or school for their various problems during the study period. Also, some of the workers who attended to the families in the control group had received IY^®^ training, which suggests that they might have used these strategies with some parents in the control group, as was noticed in a survey carried out among Finnish IY Group Leaders [67]. The survey reported that 90% of the workers stated that their whole way of working changed after IY group leader training, becoming more empathic and client-focused, and they were able to support parenting more broadly and concretely. This being the case, the control group condition was a suboptimal control.

## Conclusions

Despite the study limitations, our results suggest that IY^®^ parent groups are an acceptable and effective intervention for child conduct problems in the short term in families with CPS contact, and that it is possible to do long-term research in this subgroup of parents and children. Even though there were more positive changes in child behaviour in the intervention group from pre-assessment to post-assessment compared with the control group in some of the domains, the intervention effects were not sustained to the one-year follow-up. In order for parents to maintain the new, effective parenting methods, they would need more support and constant reminders also after the intervention. This can be challenging to provide since parents in CPS often face multiple high-level stressors in their lives in addition to child behaviour problems, thus also having other issues that need to be addressed effectively. It is therefore important to identify all risk factors in the lives of these families and to create a long-term plan with a multidisciplinary team in order to better target services to the needs of the families. Still, it is noteworthy that when looking at the whole study group (combined control and intervention) it seemed that the child conduct problems decreased from baseline to follow-up.

Children involved in CPS are the most vulnerable group of children due to high prevalence of problem behaviours and poor academic performance—doubling the risks for life course trajectories leading to multiple problems such as substance use, criminal behaviour, mental disorders, poor health, academic underachievement, unemployment and elevated mortality. For this reason, it is important to determine the services that work best for these families and children.

## Data Availability

The data that support the findings of this study are available from the Finnish institute for health and welfare, but restrictions apply to the availability of these data, which were used under license for the current study, and so are not publicly available. Data are however available from the authors upon reasonable request and with permission of the Finnish institute for health and welfare. All analyses were conducted using SPSS software version 26. The syntaxes for the analysis are available upon request.
